# Presence of SARS-CoV-2 in a Cornea Transplant

**DOI:** 10.3390/pathogens10080934

**Published:** 2021-07-24

**Authors:** Myriem Otmani Idrissi, Jean-Pierre Baudoin, Anne-Line Chateau, Sarah Aherfi, Marielle Bedotto-Buffet, Alain Latil, Hubert Lepidi, Jacques Chiaroni, Christophe Picard, Jean-Louis Mege, Bernard La Scola, Soraya Mezouar

**Affiliations:** 1 Aix-Marseille University, MEPHI, IRD, APHM, 13005 Marseille, France; myriemotm@gmail.com (M.O.I.); jean-pierre.baudoin@ap-hm.fr (J.-P.B.); sarash.aherfi@univ-amu.fr (S.A.); marielle.bedotto@univ-amu.com (M.B.-B.); hubert.pelidi@univ-amu.fr (H.L.); jean-louis.mege@univ-amu.fr (J.-L.M.); 2 IHU-Méditerranée Infection, 13005 Marseille, France; 3 EFS Provence Alpes Côte d’Azur Corse, 13005 Marseille, France; Anne-line.CHATEAU@efs.sante.fr (A.-L.C.); jacques.chiaroni@univ-amu.fr (J.C.); christophe.picard@efs.sante.fr (C.P.); 4 CH d’Antibes-Juan Les Pins, 06160 Antibes, France; alain.latil@efs.sante.fr; 5 Aix-Marseille University, EFS, CNRS, ADES, 13005 Marseille, France

**Keywords:** cornea, SARS-CoV-2, COVID-19

## Abstract

Background: The SARS-CoV-2 pandemic has impacted tissue transplantation procedures since conjunctivas were found to be associated with coronavirus infection. Here, we investigated infection of a cornea graft from a COVID-19-positive donor. Methods: In order to evaluate the presence of SARS-CoV-2 in the cornea graft we first carried out a qRT-PCR and then we investigated the presence of SARS-CoV-2 by fluorescence and electron microscopy. Conclusions: Although the cornea graft was found to be negative by qRT-PCR, we were able to show the presence of SARS-CoV-2 in corneal cells expressing the SARS-CoV-2 receptor, ACE2. Taken together, our findings may have important implications for the use of corneal tissue in graft indications and open the debate on SARS-CoV-2 transmissibility.

## 1. Introduction, Results and Discussion

The SARS-CoV-2 pandemic has radically impacted ophthalmology practices, particularly tissue transplantation procedures, as conjunctivas have been identified to be associated with coronavirus infection [1]. Although no SARS-CoV-2 has been detected via qRT-PCR in ocular tissue, some rare cases of conjunctivitis were reported with positive SARS-CoV-2 qRT-PCR results from conjunctival secretions and tears [1]. Moreover, SARS-CoV-2 infection of macaques was reported through the conjunctival pathway [2], suggesting that SARS-CoV-2 used the conjunctival route of transmission. Finally, the expression of angiotensin-converting enzyme-2 (ACE2) was found on corneal and conjunctival cells [3], suggesting that the cornea could be a target tissue for SARS-CoV-2 infection. The nucleocapsid protein antigen of SARS-CoV-2 was found intracellularly in the ocular tissues of a patient without ocular signs two months after infection [4]. Here, we hypothesize that without ocular clinical signs, SARS-CoV-2 may be present in corneal grafts.

**Figure 1:**
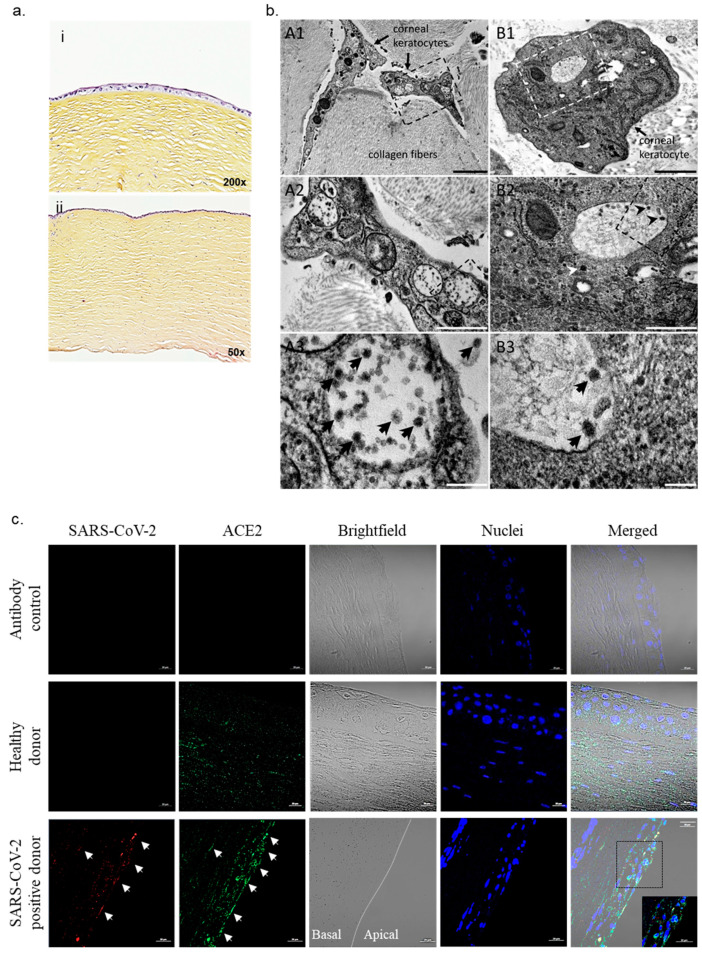
SARS-CoV-2 in corneal transplant. (**a**) Hematoxylin and eosin labeling in corneal tissue from a tissue donor with a positive PCR test for SARS-CoV-2 shows the absence of inflammation or lesions. (**b**) Transmission electron microscopy of corneal tissue. (A1–A3) Different magnification views of keratocytes embedded in collagen stromal matrix. A1: Low-magnification view of two cellular regions and of collagen fibers cut tangentially or transversally. A2: Zoomed-in boxed region in (A1), with three vacuoles filled with virus-like particles. A3: Zoomed-in boxed region in (A2), with intravacuolar and extracellular SARS-CoV-2-like particles. (B1–B3): Different magnification views of a keratocyte cell body embedded in collagen stromal matrix. B1: Low-magnification view of a keratocyte cell body with the Golgi apparatus and endoplasmic reticulum, mitochondria and vacuoles. B2: Zoomed-in boxed region in (B1), with Golgi vesicles (**bottom**), granular endoplasmic reticulum (**top-left**), mitochondria and a vacuole filled with virus-like particles (**center**). B3: Zoomed-in boxed region in (B2), with SARS-CoV-2-like particles in a vacuole. SARS-CoV-2-like particles (arrows in B2, A3 and B3) were pleomorphic with a dense core, a corona with spikes and a mean diameter of 56 ± 10 nm. Scale bars: A1, 2 µm; A2, 1 µm; A3, 200 nm; B1, 1 µm; B2, 500 nm; B3, 100 nm. (**c**) Immunostaining of corneal tissue from postmortem donors with and without COVID-19-positive PCR for spike protein from SARS-CoV-2 (red) and ACE2 (green) indicated with white arrows, with nuclei in blue. Scale bar: 20 µM.

We report a case of corneas collected postmortem from a SARS-CoV-2-positive donor that had no signs of eye disease, such as conjunctivitis. The donor, an 88-year-old woman with no known infection history of SARS-CoV-2, was admitted to the hospital for abdominal pain in the context of colon cancer. The patient had no evidence of upper respiratory infection, and a qRT-PCR investigation of nasopharyngeal samples performed at the admission of the patient was negative. The patient died two days later, following the evolution of carcinoma. In accordance with the French regulations for tissue donation (Agence de Biomedecine), a new postmortem RT-PCR test on nasopharyngeal samples, serology (IgG–IgM antibodies, BIOSYNEX, Brant, France) and total antibody tests (ECLIA Cobas, Roche Technologies, Boulogne Billancourt, France) were performed with a positivity for SARS-CoV-2. qRT-PCR investigation on both media containing the corneal grafts and corneal tissues was negative for SARS-CoV-2. Histological examination of corneal grafts showed no lesions, inflammation or tissue damage (epithelium, stroma, endothelium) (Figure 1a). These results, from tests traditionally performed in routine clinical practice, did not show the presence of SARS-CoV-2 in corneal tissue from COVID-19-positive organ donors.

Regardless of the negative qRT-PCR, we sought to determine whether there was SARS-CoV-2 within the tissue. As histopathological evaluation of positive COVID-19 patients was crucial [5], we turned to electron microscopy since it has been reported to have some utility in identifying viral particles. We observed numerous virus-like particles localized to large vacuoles in the two corneal grafts (Figure 1b). SARS-CoV-2-like particles were pleomorphic with or without peripheral corona fibers, with a mean diameter of 56 ± 10 nm (Figure 1b). Although no conjunctivas and no tissue damage were reported, searching for other viruses involved in keratitis/conjunctivitis appeared pertinent [6]. Testing for herpes simplex virus, cytomegalovirus, enterovirus and adenovirus was negative. We conducted immunofluorescence staining and confirmed the presence of SARS-CoV-2 in corneal grafts (Figure 1c). At the apical part of the cornea consisting of epithelial cells, we observed the presence of SARS-CoV-2 in corneal cells expressing ACE2 compared to corneal grafts from postmortem donors devoid of SARS-CoV-2 infection.

Taken together, this study depends on the use of PCR on the donor tissue to test for the qualification of corneal grafts and the risk of transmission. No lesion or inflammation was observed, suggesting a quiescent state of the virus in the corneal cells, as observed for other types of viruses [6]. Although no studies have reported posttransplant COVID-19 infection thus far, positive SARS-CoV-2 RNA from ocular tissues has been reported to be associated with conjunctivitis [7]. In these conditions, and in the absence of viricidal activity in culture media, the European Centre for Disease Prevention and Control recommends using at least one exposure of ocular tissue to a biocidal agent in their donor protocol. The emergence of studies illustrating the presence of SARS-CoV-2 in corneas should be considered in the regulation of tissue transplantation guidelines, even if this option remains debated [8,9].

Finally, clinical evaluation of patients has been deeply changed during the COVID-19 pandemic [10,11]. Here, we reported that the cornea can be infected by SARS-CoV-2. This case, with direct observation of the virus, and another with viral protein detection, suggests infection in the cornea, isolated from acute symptomatic SARS-CoV-2 infection. Taken together, our findings may have important implications for the validation and the use of tissue corneal grafts, opening the debate on SARS-CoV-2 transmissibility.

## 2. Materials and Methods

### 2.1. Cornea Tissue

Corneal tissues were obtained from one healthy donor and one postmortem donor reported as carrying SARS-CoV-2 and preserved in Cornea Max medium (Eurobio, Les Ulis, France).

### 2.2. RT-PCR Analysis

Viral RNA was extracted from tissue using a NucleoSpin^®^kit (Macherey-Nagel, Hoerdt, France) and SARS-CoV-2 detection was evaluated by qRT-PCR using a SuperScript^TM^ III PlatinumTM Kit (Life Technologies, Carlsbad, CA, USA). Thermal cycling was achieved at 55 °C for 10 min for reverse transcription, followed by 95 °C for 3 min and then 45 cycles at 95 °C for 15 s and 58 °C for 30 s using a LightCycler-480 system (Roche, Meylan, France). We investigated the E and N genes for the detection of SARS-CoV-2 as previously described [12]. Primers and probes were designed against the E (forward: ACAGGTACGTTAATAGTTAATAGCG, reverse: ATATTGCAGCAGTACGCACACA, probes: FAM-ACACTAGCCATCCTTACTGCGCTTCG-QSY) and N (forward: GACCCCAAAATCAGCGAAAT, reverse: TCTGGTTACTGCCAGTTGAATCTG, probes: FAM-ACCCCGCATTACGTTTGGTGGACC-QSY) genes. RT-PCR was performed for the detection of adenovirus, enterovirus, cytomegalovirus and herpes simplex virus as previously described [13,14,15,16].

### 2.3. Histological Analysis

Corneal tissues (5 µM sections) were labeled using hematoxylin–eosin–saffron coloration and observed using an optical microscope. For immunostaining, slides were incubated in a saturation solution containing 3% bovine serum albumin (Eurobio, #5154-0006) and 0.1% Tween-20 (Sigma-Aldrich, #P9416, Saint-Quentin-Fallavier, France) diluted in phosphate-buffered saline (Life Technologies, Gibco, #14190144). The slides were incubated with anti-ACE2 (1:500, antibodies-online, #AA-599-649) and anti-SARS-CoV-2 spike protein (1:500, Thermo Fisher, #PA581795, Carlsbad, CA, USA) and nuclei were counterstained with 4′,6-diamidino-2-phenylindole (Invitrogen, #D1306, Carlsbad, CA, USA). Next, the sections were incubated with goat anti-rabbit (Thermo Fisher, #A11010) and donkey anti-mouse IgG secondary antibodies (Thermo Fisher, #A11003). Secondary antibodies alone were used for the detection of nonspecific background. After washing, sections were analyzed using an LSM800 Airyscan confocal microscope (Zeiss, 63×, Jena, Germany).

### 2.4. Transmission Electron Microscopy

Cornea tissues were fixed with glutaraldehyde 2.5% (Biovalley, #16210, Nanterre, France). Resin embedding was microwave-assisted with a BiowavePro^+^ (Pelco, Roseville, CA, USA). Samples were washed with a 0.2 M saccharose (Sigma-Aldrich, #1076870250)/0.1 M sodium cacodylate (Sigma-Aldrich, #70114) buffer and postfixed with 1% OsO4 (Sigma-Aldrich, #201030) diluted in 0.2 M potassium hexa-cyanoferrate (III) (Sigma-Aldrich, #P8131)/0.1 M sodium cacodylate buffer. After washes with distilled water, samples were gradually dehydrated by successive 50%, 70% and 96% ethanol baths. Substitution with hard grade LR-White resin (Polysciences, Sigma-Aldrich, #94188-59-7) was achieved by incubations with a mixture of 100% LR-White resin and 96% ethanol in a 2:1 ratio, incubations with 100% LR-White resin and completed with samples in 100% LR-White resin overnight under vacuum. Resin heat curing was achieved by polymerization for 72 h at 60 °C. Ultrathin 70 nm sections were cut using a UC7 ultramicrotome (Leica, Edinburgh, UK) and placed on HR25-300 mesh copper/rhodium grids (TAAB, Berks, UK). Sections were contrasted according to Reynolds^20^. Pictures were obtained on a Morganii 268D (Philips, Salina, CA, USA) transmission electron microscope operated at 80 keV and equipped with a MegaView3 camera.

## Data Availability

The data presented in this study are available on request from the corresponding author. The data are not publicly available due to ethical restrictions.
